# Lead Optimization of Dehydroemetine for Repositioned Use in Malaria

**DOI:** 10.1128/AAC.01444-19

**Published:** 2020-03-24

**Authors:** Priyanka Panwar, Kepa K. Burusco, Muna Abubaker, Holly Matthews, Andrey Gutnov, Elena Fernández-Álvaro, Richard A. Bryce, James Wilkinson, Niroshini Nirmalan

**Affiliations:** a Environment and Life Sciences, University of Salford, Greater Manchester, United Kingdom; b Division of Pharmacy and Optometry, School of Health Sciences, Faculty of Biology, Medicine and Health, Manchester Academic Health Science Centre, University of Manchester, Manchester, United Kingdom; c Keele University, Newcastle-under-Lyme, Staffordshire, United Kingdom; d Chiroblock GMBH, Wolfen, Germany; e GlaxoSmithKline, Diseases of the Developing World Medicines Development Campus, Tres Cantos, Spain

**Keywords:** malaria, antimalarial drug interactions, SYBR green flow cytometry, emetine, dehydroemetine, drug discovery, repositioning

## Abstract

Drug repositioning offers an effective alternative to *de novo* drug design to tackle the urgent need for novel antimalarial treatments. The antiamoebic compound emetine dihydrochloride has been identified as a potent *in vitro* inhibitor of the multidrug-resistant strain K1 of Plasmodium falciparum (50% inhibitory concentration [IC_50_], 47 nM ± 2.1 nM [mean ± standard deviation]). Dehydroemetine, a synthetic analogue of emetine dihydrochloride, has been reported to have less-cardiotoxic effects than emetine. The structures of two diastereomers of dehydroemetine were modeled on the published emetine binding site on the cryo-electron microscopy (cryo-EM) structure with PDB code 3J7A (P. falciparum 80S ribosome in complex with emetine), and it was found that (−)-*R*,*S*-dehydroemetine mimicked the bound pose of emetine more closely than did (−)-*S*,*S*-dehydroisoemetine. (−)-*R*,*S*-dehydroemetine (IC_50_ 71.03 ± 6.1 nM) was also found to be highly potent against the multidrug-resistant K1 strain of P. falciparum compared with (−)-*S*,*S*-dehydroisoemetine (IC_50_, 2.07 ± 0.26 μM), which loses its potency due to the change of configuration at C-1′. In addition to its effect on the asexual erythrocytic stages of P. falciparum, the compound exhibited gametocidal properties with no cross-resistance against any of the multidrug-resistant strains tested. Drug interaction studies showed (−)-*R*,*S*-dehydroemetine to have synergistic antimalarial activity with atovaquone and proguanil. Emetine dihydrochloride and (−)-*R*,*S*-dehydroemetine failed to show any inhibition of the hERG potassium channel and displayed activity affecting the mitochondrial membrane potential, indicating a possible multimodal mechanism of action.

## Abstract (precis)

Drug repositioning offers an effective alternative to *de novo* drug design to tackle the urgent need for novel antimalarial treatments. The antiamoebic compound emetine dihydrochloride has been identified as a potent *in vitro* inhibitor of the multidrug-resistant strain K1 of Plasmodium falciparum (50% inhibitory concentration [IC_50_], 47 nM ± 2.1 nM [mean ± standard deviation]). Dehydroemetine, a synthetic analogue of emetine dihydrochloride, has been reported to have less-cardiotoxic effects than emetine.

## TEXT

Malaria presents a huge burden on the economic development of countries of endemicity (1). In 2017, WHO reported 219 million malaria cases globally, with an estimated 435,000 deaths, occurring mostly among African children. The 20th century witnessed the development of a range of antimalarials, including quinine alternatives like mepacrine, chloroquine, and primaquine, antifolates like sulfadoxine and pyrimethamine, and artemisinin (2–7). However, the emergence of resistance against all known classes of antimalarial drugs, including artemisinin chemotherapy, has warranted research into the development of new drugs with novel targets against the parasite (8, 9). Drug discovery is hindered by long development timelines, high attrition rates, and soaring research and development costs (10). Antimalarial chemotherapy has predominantly relied on compounds based on natural products (11). Emetine dihydrochloride hydrate, a natural product alkaloid derived from Psychotria ipecacuanha, was superseded by the introduction as an antiamoebic drug of a safer drug, metronidazole, by the 1970s (12). Repositioning screens carried out at the University of Salford, United Kingdom, identified emetine dihydrochloride as having potent, nanomolar *in vitro* antimalarial efficacy in the multidrug-resistant (MDR) Plasmodium falciparum parasite strain K1. The comparatively significant difference in *in vitro* antiprotozoan efficacies (50% inhibitory concentration [IC_50_] of 47 ± 2.1 nM [mean ± standard deviation] in P. falciparum compared to IC_50_ of 26.8 ± 1.27 μM in Entamoeba histolytica) dictate that the safety profile for its repositioned use as an antimalarial could be different (13, 14). The pleiotropic natural product drug has been reported to have antiviral and anticancer properties, including recent reports of interrupting viral replication and cell entry for Zika and Ebola viruses (15). The 40S ribosomal subunit of the eukaryotic 80S ribosome was recently reported to be the site of action of emetine and is available as a cryo-electron microscopy (cryo-EM) structure with PDB code 3J7A (16). The definition of the target binding site for emetine enables a chimeric approach to refine, using rational design, a drug discovered through repositioning.

**FIG 1:**
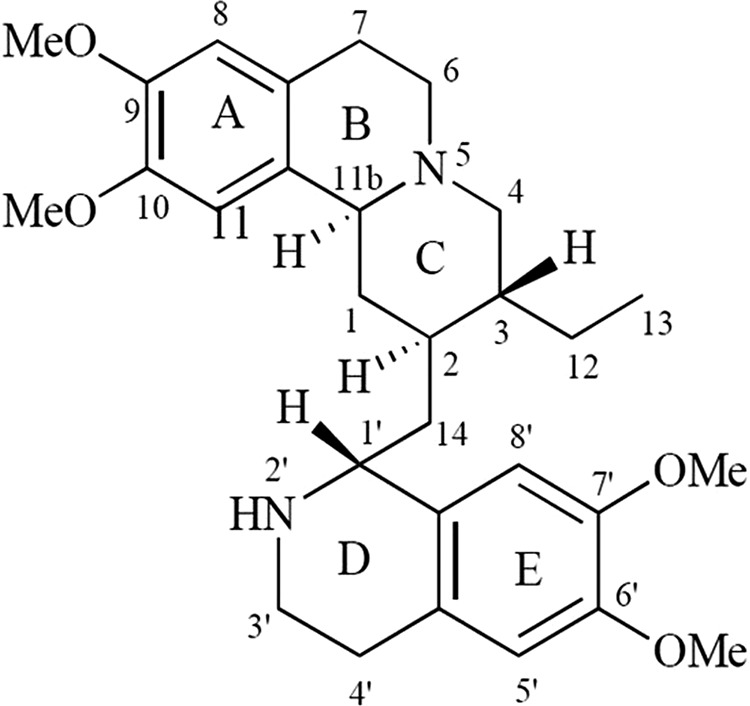
Structure of emetine hydrochloride.

The (*R*) configuration at C-1′ and the presence of secondary nitrogen at position 2′ are important for emetine’s biological activity (Fig. 1). Indeed, the (*S*) configuration at C-1′ (isoemetine) or the substitution of the secondary amine results in loss of activity. Even if the asymmetry at carbons 2 and 3 is lost with unsaturation at position 2-3 (2,3-dehydroemetine), the biological activity is retained (17). In 1980, a study conducted on the cross-resistance of emetine-resistant mutants of Chinese hamster ovary cells to related compounds found that the distance between the two aromatic rings and the angle between the nucleophilic element, such as nitrogen, and the rings were essential for biological activity (18).

Emetine in its crude form has been in use since 1658 for the treatment of dysentery and was first brought to Europe from Brazil by Piso (19). Powdered ipecacuanha introduced in Mauritius in 1858 reduced the annual death rate from severe dysentery from 10 to 18% to 2% (20). Generalized muscle weakness, vomiting, and cardiotoxicity were the side effects with prolonged use of emetine (21, 22). A less emetic and safer synthetic analogue of emetine which could be given as a resinate, 2,3-dehydroemetine, was introduced in 1959 (23). At high doses, the electrocardiographic changes were similar but were less marked and of shorter duration. The observed changes in heart conduction, contractility, automaticity, and electrocardiogram (ECG) abnormalities caused by emetine and 2,3-dehydroemetine could be due to their effect on membrane permeability to Na^+^, K^+^, and Ca^++^ ions. Dehydroemetine has been reported to have less-cardiotoxic effects than emetine for the treatment of amoebic liver abscesses (24–26).

Previous studies have found that dehydroemetine was eliminated faster from the body and more rapidly from the heart than from the liver, while the reverse was found to be true for emetine (27). Based on the anecdotal evidence, two diastereomers of dehydroemetine, (−)-*R*,*S*-dehydroemetine and (−)-*S*,*S*-dehydroisoemetine, were synthesized. Molecular modeling tools (28, 29) were used to predict the activities of the diastereomers and their potencies against the multidrug-resistant K1 strain of P. falciparum.

The parasites are transmitted from their mammalian hosts to mosquito vectors through mature *Plasmodium* gametocytes, and hence, the reinfection cycle could be broken with the use of transmission-blocking antimalarials (30). The cross-resistance and gametocidal activities of emetine dihydrochloride and its synthesized analogues were tested by GSK in a bid to determine the potential of these compounds as transmission-blocking drugs.

A number of studies have reported that dehydroemetine and emetine are potent inhibitors of protein synthesis (17, 31). It has also been reported that emetine affects the myocardium in a dose-dependent manner (32). Drugs inhibiting the cardiac potassium ion channel encoded by the human ether-a-go-go-related gene (hERG) can prolong the QT interval and cause a dangerous cardiac arrhythmia, Torsades de pointes, which has hampered a number of drug discovery and development projects (33). In this study, we tested the activity of emetine dihydrochloride and its two synthetic analogues against the hERG potassium channel. The three compounds were also tested for any effect on mitochondrial membrane potential (MMP), as unpublished data from previous studies conducted by our group predicted atovaquone-like activity affecting MMP by the parent compound, emetine.

The preference for combinatorial regimes over monotherapy for the treatment of malaria has affected the drug discovery pipeline in a crucial way (34). Newer drug candidates have been tested for synergistic activities with existing antimalarial treatments for dose reduction to improve therapeutic and safety profiles. Furthermore, combinatorial regimes expand the effective life of the antimalaria drugs by delaying the emergence of resistance (35, 36).

Chou and Talalay developed a method based on the argument that the issue of synergy is more physiochemical rather than statistical in nature and employed the law of mass action to derive a median-effect equation where additivity could be defined using the resulting combination index (CI = 1), with antagonism and synergism defined as >1 and <1, respectively (37). CalcuSyn software based on the complex algorithms for median-effect analysis allows automation and eliminates subjectivity during data analysis (37). We have previously demonstrated the use of CalcuSyn as a reliable method to define antimalarial drug interactivity for combinatorial regimes (38).

Hence, to reduce dose-dependent side effects, combinatorial partner drugs showing synergistic activity were sought. (−)-*R*,*S*-Dehydroemetine was taken forward for drug interaction studies as it was found to be highly potent against the K1 strain of P. falciparum. Atovaquone and proguanil were first evaluated to test the efficacy of the method, as the two drugs are known to exhibit synergism (39). The methodology was then applied to evaluate combinatorial partner drugs for the potent antimalarial candidate (−)-*R*,*S*-dehydroemetine.

## RESULTS

### Molecular modeling of ligand interactions with P. falciparum 80S ribosome.

In order to explore the structural basis of the relative inhibitory activities of (−)-*R*,*S*-dehydroemetine and (−)-*S*,*S*-dehydroisoemetine, we predicted and compared their molecular interactions with the P. falciparum 80S ribosome using computational docking. As the receptor structure, we used the recently solved cryo-EM structure of the 80S ribosome of P. falciparum, determined to a resolution of 3.2 Å (16). The electron density corresponding to the bound emetine is located in the E site of the ribosomal small subunit, i.e., the P. falciparum 40S ribosome (*Pf*40S); this E site, a binding site that pactamycin also recognizes in the bacterial 30S subunit, is at the interface between 18S rRNA helices 23, 24, and 45 and the C terminus of protein uS11.

**FIG 2:**
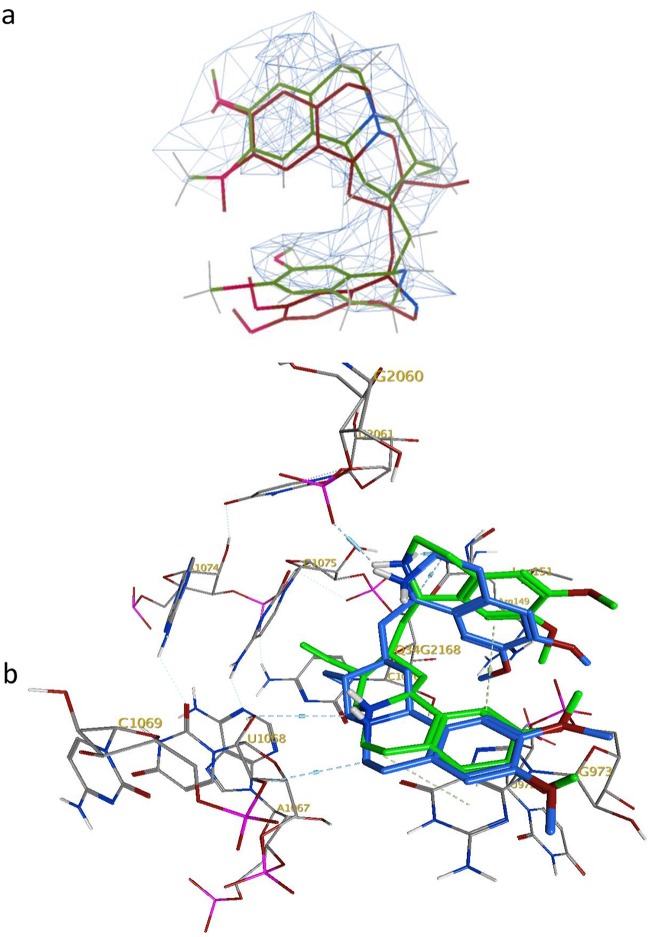
(a) Overlay of the docked pose of emetine (green) with its enantiomer present in the cryo-EM structure (maroon); observed electron density envelope is also shown (wireframe surface with contour level 0.1542 electrons/Å̂3 [3.50 RMSD]). (b) Interactions of docked emetine (green) with *Pf*40S residues and comparison with previously modeled interactions of its enantiomer (blue) (16).

On closer examination of the published cryo-EM structure of the complex, the ligand structure originally modeled into the density erroneously corresponds to the nonnatural enantiomer of emetine, (1*S*,2*R*,3*S*,11b*R*)-emetine. This modeled structure was not the ligand employed in the associated cryo-EM experiments (16). Therefore, we docked emetine, i.e., the (1*R*,2*S*,3*R*,11b*S*) structure, into the identified *Pf*40S binding site using MOE-Dock (2016; Chemical Computing Group, Inc., Montreal, QC, Canada). The docked emetine geometry maps well into the electron density envelope (shown at a contour level of 0.1542 electrons/Å̂3 [3.50 root mean square deviation {RMSD}] in Fig. 2a); the bound pose broadly follows the twisted U shape conformation of the observed electron density, with improved alignment in comparison to the previously published nonnatural emetine enantiomer geometry (Fig. 2b).

**FIG 3:**
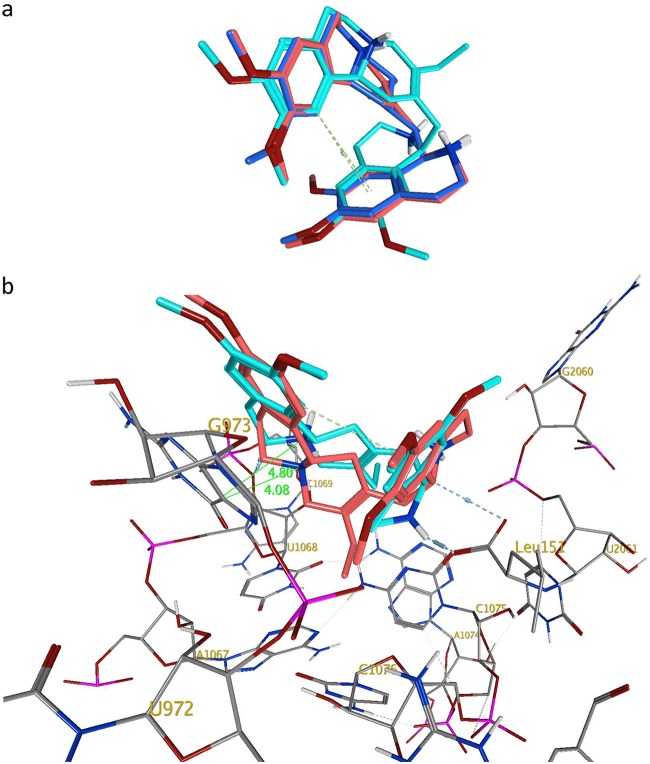
(a) Overlay of docked poses of (−)-*R*,*S*-dehydroemetine (red) and (−)-*S*,*S*-dehydroisoemetine (cyan) with emetine (blue). (b) Interactions of (−)-*R*,*S*-dehydroemetine (red) and (−)-*S*,*S*-dehydroisoemetine (cyan) with the *Pf*40S binding site. Distances (dotted lines) in Å.

In this U shape, an intermolecular T-shaped π-stacking interaction is observed between the two cyclic systems of emetine, i.e., benzo[a]quinolizine rings A/B/C and isoquinoline rings D/E (Fig. 1 and 2a). The docked emetine also forges a number of comparable interactions with the *Pf*40S subunit as its modeled enantiomer (Fig. 2b), with a key π-stacking interaction between the A/B/C rings of emetine and the purine ring of G973 of h23 (16). The locations of the emetine secondary and tertiary amines are broadly similar (within 1 to 2 Å), allowing hydrogen bonding interactions with a backbone oxygen atom of U2061 (h45) and the 2′-hydroxyl group of U1068 (h24), respectively (Fig. 2b). The tertiary amine also forms a salt bridge interaction with the carboxylate side chain of C-terminal residue Leu151 of uS11 (Fig. 3b). This interaction was not highlighted in the cryo-EM study and is a consequence of modeling the natural emetine geometry into the E site.

Subsequently, the two diastereomers of emetine, (−)-*R*,*S*-dehydroemetine and (−)-*S*,*S*-dehydroisoemetine, were docked in turn into the emetine binding region of the *Pf*40S subunit. We found that the preferred docked pose of the (−)-*R*,*S*-dehydroemetine adopts the familiar U-shaped conformation, superimposing rather closely onto the bound pose of emetine (Fig. 3a). The docking scores are correspondingly similar for emetine, with a London dG value (a scoring function in molecular docking that estimates the binding free energy of the ligand from a given pose) of −7.2 kcal/mol, and (−)-*R*,*S*-dehydroemetine, which has a dG score of −7.3 kcal/mol. As observed for emetine, (−)-*R*,*S*-dehydroemetine forms the π−π stacking interaction with the G973 pyrimidine ring and polar interactions with U2061, U1068, and Leu151 (Fig. 3b).

**FIG 4:**
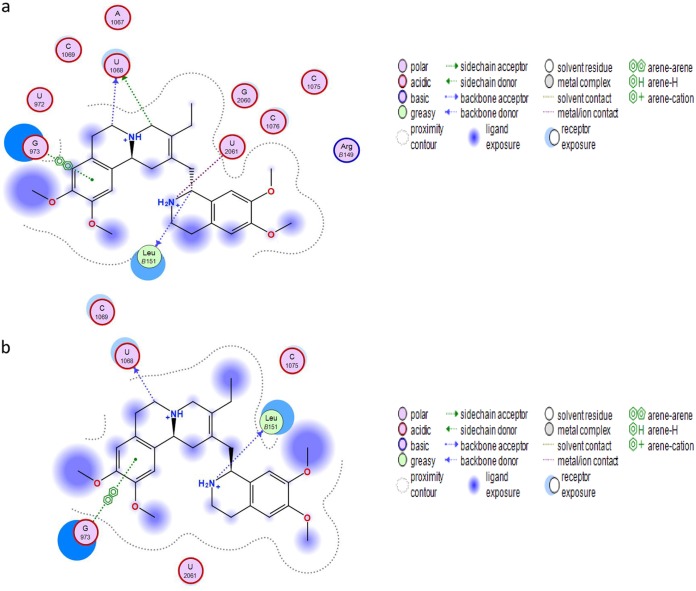
(a) Docking through MOE showing the binding site residues for (−)-*R*,*S*-dehydroemetine molecule. (b) Docking through MOE showing the binding site residues for (−)-*S*,*S*-dehydroisoemetine molecule.

However, (−)-*S*,*S*-dehydroisoemetine docks into the binding site with a lower dG score of −6.5 kcal/mol and does not overlay in conformation with emetine or (−)-*R*,*S*-dehydroemetine so readily (Fig. 3a). The particular stereochemical configuration of (−)-*S*,*S*-dehydroisoemetine appears to result in its secondary amine being more distant from the E-site residues of *Pf*40S. Consequently, this amine N^…^O U2061 distance extends by 0.8 Å in proceeding from the R to the S isomer (Fig. 3b). The tertiary amine interaction with U1068 is maintained, however, as is the interaction with the terminal carboxylate of Leu151 (Fig. 3b). The π stacking interaction with G973 is also present, but at a slightly larger distance between planes, increased by ∼0.7 Å. Figure 4a and b show the predicted binding site residues for (−)-*R*,*S*-dehydroemetine and (−)-*S*,*S*-dehydroisoemetine.

### Experimental determination of IC_50_s to test activity against the K1 strain of P. falciparum.

**FIG 5:**
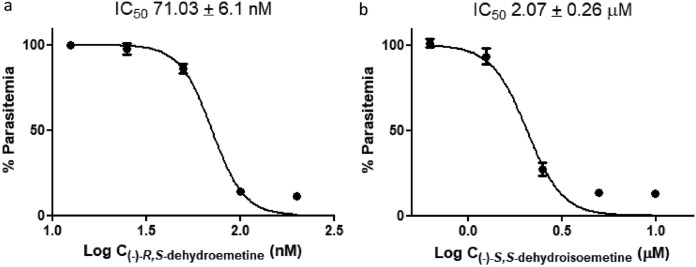
(a) The effective dose of (−)-*R*,*S*-dehydroemetine (dose range tested in 2-fold serial dilutions from 12.5 nM to 200 nM) on P. falciparum K1 infection after an incubation period of 72 h using SYBR green-based plate reader assay. (b) The effective dose of (−)-*S*,*S*-dehydroisoemetine (dose range tested in 2-fold serial dilutions from 0.625 μM to 10 μM) on P. falciparum K1 infection after an incubation period of 72 h using SYBR green-based plate reader assay. The experiments were performed thrice with each concentration of (−)-*R*,*S*-dehydroemetine and (−)-*S*,*S*-dehydroisoemetine (tested in triplicates). Data were analyzed using GraphPad prism. Error bars show standard deviations.

Based on the anecdotal evidence, two synthetic analogues of emetine dihydrochloride, (−)-*R*,*S*-dehydroemetine and (−)-*S*,*S*-dehydroisoemetine, were synthesized. Experiments to test drug efficacy were set up, and (−)-*R*,*S*-dehydroemetine was tested in 2-fold serial dilutions from 12.5 nM to 200 nM. A dose-response experiment was set up on synchronized ring stage cultures of the K1 strain of P. falciparum to be read at 72 h. The IC_50_ of (−)-*R*,*S*-dehydroemetine was observed to be 71.03 ± 6.1 nM (Fig. 5a). (−)-*S*,*S*-dehydroisoemetine was tested in 2-fold serial dilutions from 0.625 μM to 10 μM. The IC_50_ was observed to be 2.07 ± 0.26 μM (Fig. 5b). The results shown in Fig. 5 are derived from representative experiments performed thrice, with each concentration of (−)-*R*,*S*-dehydroemetine and (−)-*S*,*S*-dehydroisoemetine tested in triplicates.

### Time course analysis for determination of speed of action of (−)-*R*,*S*-dehydroemetine and (−)-*S*,*S*-dehydroisoemetine.

Accurate determination of the parasite killing rate in response to treatment is crucial in the development of drugs against P. falciparum, as chemotherapy remains the primary element in the control of malaria. The speed of action of compounds on the viability of parasites is difficult to measure through traditional techniques (40). It is important to identify new drugs with rapid parasite-killing kinetics early in the drug development process. Besides rapid relief of symptoms, a fast-acting drug also helps to curtail the mutations causing the development of new mechanisms of drug resistance. In 2012, Tres Cantos developed a labor-intensive low-throughput assay taking up to 28 days to determine *in vitro* the parasite reduction ratio (PRR) and the presence or absence of a lag phase in response to a drug (41). The method used in this study to differentiate between fast- and slow-acting compounds gives initial results in 4 to 7 days (42).

**FIG 6:**
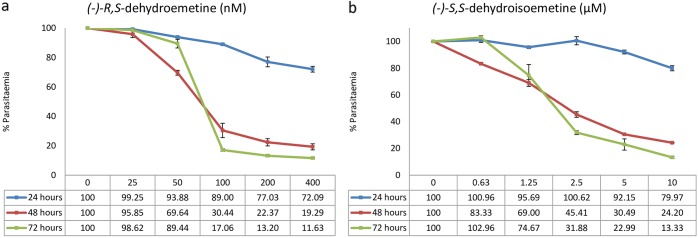
Time course analysis through IC_50_ speed assay using unsynchronized cultures of Plasmodium falciparum. The graphs show that IC_50_s could not be reached within 24 h. (−)-*R*,*S*-dehydroemetine and (−)-*S*,*S*-dehydroisoemetine reached the IC_50_ by 48 h. Error bars represent the standard errors of the results from experiments performed twice with each concentration of (−)-*R*,*S*-dehydroemetine and (−)-*S*,*S*-dehydroisoemetine (tested in triplicates). (a) (−)-*R*,*S*-dehydroemetine at concentrations of 25, 50, 100, 200, and 400 nM at 24 h, 48 h, and 72 h. (b) (−)-*S*,*S*-dehydroisoemetine at concentrations of 0.63, 1.25, 2.5, 5, and 10 μM at 24 h, 48 h, and 72 h.

The IC_50_ speed assay was performed for (−)-*R*,*S*-dehydroemetine and (−)-*S*,*S*-dehydroisoemetine (with concentrations of 25 nM, 50 nM, 100 nM, 200 nM, and 400 nM and 0.63 μM, 1.25 μM, 2.5 μM, 5 μM, and 10 μM, respectively) using unsynchronized cultures of P. falciparum. The ratios of 24-h IC_50_s to 72-h IC_50_s for (−)-*R*,*S*-dehydroemetine and (−)-*S*,*S*-dehydroisoemetine could not be determined, as the IC_50_s could not be reached within 24 h. Both compounds only achieved 50% inhibition at the previously defined IC_50_s after 48 h of exposure, indicating that the isomers have delayed action against the multidrug-resistant K1 strain of P. falciparum. Figure 6 shows the results from the IC_50_ speed assays for (−)-*R*,*S*-dehydroemetine and (−)-*S*,*S*-dehydroisoemetine [error bars in the figure represent the standard errors from experiments performed twice with each concentration of (−)-*R*,*S*-dehydroemetine and (−)-*S*,*S*-dehydroisoemetine, tested in triplicates].

### Stage-specific profiling of (−)-*R*,*S*-dehydroemetine and (−)-*S*,*S*-dehydroisoemetine using synchronized cultures of Plasmodium falciparum.

The two isomers of 2,3-dehydroemetine were tested on synchronous cultures [(−)-*R*,*S*-dehydroemetine was tested in 2-fold serial dilutions from 132.81 nM to 8,500 nM, and (−)-*S*,*S*-dehydroisoemetine was tested in 2-fold serial dilutions from 3.13 μM to 200 μM] to determine the stage specificities of the compounds by measuring the concentration-dependent growth of schizonts and rings following incubation with the two compounds.

It was observed that (−)-*R*,*S*-dehydroemetine and (−)-*S*,*S*-dehydroisoemetine affect both the ring and trophozoite/schizont stages of the parasite. The isomers were found to be more active in the late trophozoite/schizont stage, which is consistent with the proposed protein synthesis target of the parent compound. They displayed comparatively less activity against the rings even at high concentrations. At the highest concentration tested, the growth rates for the rings and trophozoites/schizonts 24 h after the (−)-*R*,*S*-dehydroemetine postexposure wash were 50.21% and 19.75%, respectively. At the highest concentration tested, the growth rates for the rings and trophozoites/schizonts 24 h after the (−)-*S*,*S*-dehydroisoemetine postexposure wash were 70.98% and 24.67%, respectively. The lower potency against ring stage parasites may also explain in part the lag phase observed during the speed assay, where the IC_50_ was not reached after 24 h of exposure against unsynchronized cultures.

**FIG 7:**
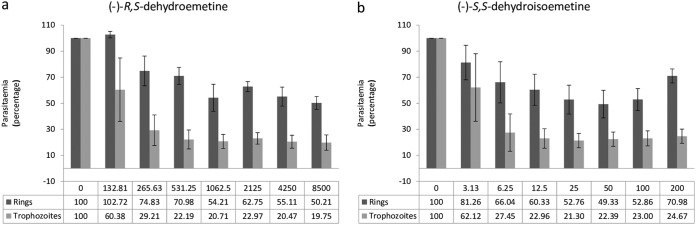
Stage-specific profiling using synchronized cultures of Plasmodium falciparum. The decrease in growth of trophozoites/schizonts was observed to be more marked than for rings. The drug effects are expressed as percentages of growth of rings relative to growth of trophozoites/schizonts 24 h after the postexposure wash. Error bars represent the standard errors of the results from experiments performed twice with each concentration of (−)-*R*,*S*-dehydroemetine and (−)-*S*,*S*-dehydroisoemetine (tested in triplicates). (a) Cultures were exposed to a serial dilution of (−)-*R*,*S*-dehydroemetine (from 132.81 nM to 8,500 nM) for 24 h. (b) Cultures were exposed to a serial dilution of (−)-*S*,*S*-dehydroisoemetine (from 3.13 μM to 200 μM) for 24 h.

Figure 7 shows the results for stage-specificity assays of (−)-*R*,*S*-dehydroemetine and (−)-*S*,*S*-dehydroisoemetine, respectively [error bars in the figure represent the standard errors from experiments performed twice with each concentration of (−)-*R*,*S*-dehydroemetine and (−)-*S*,*S*-dehydroisoemetine, tested in triplicates].

### MTT assay for cell cytotoxicity against HepG2 cells.

**FIG 8:**
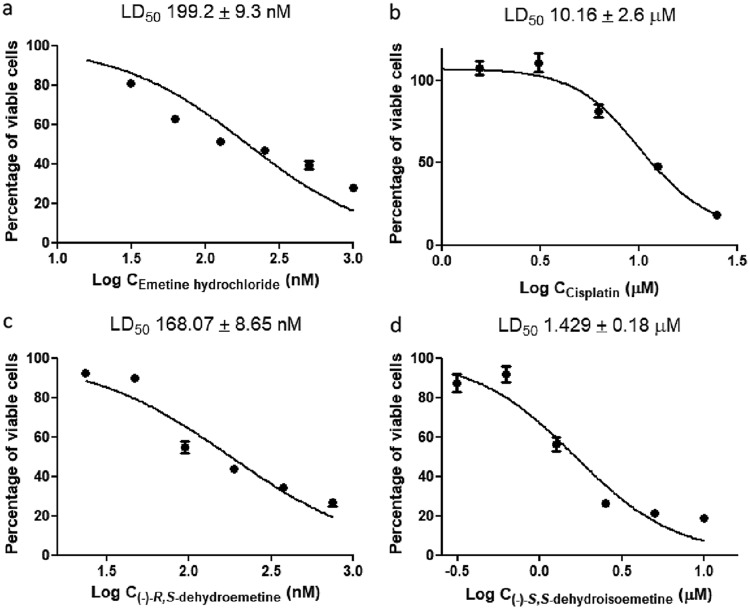
Forty-eight-hour MTT assays. Cells were seeded at 5,000 cells per well. Cell viability was determined using a standard MTT assay. Data were analyzed using GraphPad prism. Error bars represent the standard deviations of the results from experiments performed thrice with each concentration of emetine, cisplatin, (−)-*R*,*S*-dehydroemetine, and (−)-*S*,*S*-dehydroisoemetine (tested in triplicate). Forty-eight-hour MTT assays for emetine, tested in 2-fold serial dilutions from 31.25 nM to 2,000 nM (a), cisplatin, tested in 2-fold serial dilutions from 0.78 μM to 25 μM (b), (−)-*R*,*S*-dehydroemetine, tested in 2-fold serial dilutions from 11.72 nM to 750 nM (c), and (−)-*S*,*S*-dehydroisoemetine, tested in 2-fold serial dilutions from 0.312 μM to 10 μM (d).

MTT [3-(4,5-dimethyl-2-thiazolyl)-2,5-diphenyl-2H-tetrazolium bromide] assays for cell cytotoxicity were performed for (−)-*R*,*S*-dehydroemetine and (−)-*S*,*S*-dehydroisoemetine. Emetine and cisplatin were used as control drugs (Fig. 8). The plates were read at 48 h (43).

Emetine was tested in 2-fold serial dilutions from 31.25 nM to 2,000 nM, with the 50% lethal dose (LD_50_) observed to be 199.2 ± 9.3 nM. (−)-*R*,*S*-dehydroemetine was tested in 2-fold serial dilutions from 11.72 nM to 750 nM, with the LD_50_ observed to be 168.07 ± 8.65 nM. (−)-*S*,*S*-dehydroisoemetine was tested in 2-fold serial dilutions from 0.312 μM to 10 μM, with the LD_50_ observed to be 1.429 ± 0.18 μM. Cisplatin was tested in 2-fold serial dilutions from 0.78 μM to 25 μM, with the LD_50_ observed to be 10.16 ± 2.6 μM. The selectivity index, calculated as LD_50_ HepG2 cells/IC_50_ parasites (44), was 2.37 for (−)-*R*,*S*-dehydroemetine and 0.69 for (−)-*S*,*S*-dehydroisoemetine.

### Determination of cross-resistance through 3H-hypoxanthine incorporation assay.

The cross-resistance assay used relies on the parasites’ incorporation of labeled 3H-hypoxanthine, which is proportional to P. falciparum growth. *In vitro* cross-resistance of the compounds is measured as the ratio between the IC_50_ for the tested P. falciparum strain and the IC_50_ for strain 3D7A. Every replicate from a multidrug-resistant (MDR) strain involved a simultaneous determination using a 3D7A replicate to avoid any artifact linked to experimental conditions. Table 1 shows the IC_50_s of emetine dihydrochloride, (−)-*R*,*S*-dehydroemetine, and (−)-*S*,*S*-dehydroisoemetine in the sensitive P. falciparum strain (3D7A) and two resistant P. falciparum strains (Dd2 and W2). Using strain 3D7A as a reference, the ratios of *in vitro* cross-resistance of emetine dihydrochloride were found to be 1.15 for strain Dd2 and 0.77 for strain W2. The ratios for (−)-*R*,*S*-dehydroemetine in the two resistant strains, Dd2 and W2, were found to be 1.21 and 1.15, respectively.

**TABLE 1:** Analysis of results of 3H-hypoxanthine incorporation assay to determine cross-resistance

Compounds	Value for indicated P. falciparum strain(s)^*a*^							
	3D7A		Dd2		W2		Ratio of IC_50_s of:	
	IC_50_ (μM)	SD	IC_50_ (μM)	SD	IC_50_ (μM)	SD	Dd2/3D7A	W2/3D7A
Emetine dihydrochloride	0.234	0.022	0.269	0.025	0.181	0	1.15	0.77
(−)-*R*,*S*-dehydroemetine	0.146	0.003	0.177	0.014	0.168	0.005	1.21	1.15
(−)-*S*,*S*-dehydroisoemetine	1.031	0.041	ND		ND			

a *In vitro* IC_50_s of emetine dihydrochloride, (−)-*R*,*S*-dehydroemetine, and (−)-*S*,*S*-dehydroisoemetine in sensitive P. falciparum strain 3D7A and resistant P. falciparum strains Dd2 and W2, as well as ratios of *in vitro* cross-resistance of emetine dihydrochloride and (−)-*R*,*S*-dehydroemetine in both resistant strains (Dd2 and W2), using strain 3D7A as reference. ND, not determined.

The results showed that the inhibitory potencies observed for emetine dihydrochloride and (−)-*R*,*S*-dehydroemetine in both multidrug-resistant strains (Dd2 and W2) are similar to their inhibitory potencies for the sensitive strain 3D7A. These results suggest that there is no cross-resistance with any of the MDR strains tested.

### *In vitro* IC_50_s against P. falciparum male and female activated gametes.

A bioassay was performed to assess the malaria transmission-blocking potential of compounds on P. falciparum strain NF54 by estimating their ability to prevent male mature gametocytes from progressing to male microgametes or/and to inhibit female gamete activation, as indicators of gametocyte functionality. The NF54 strain was selected for its increased ability to produce gametocytes under *in vitro* conditions (45). The activation of male gametocytes into mature microgametes is evaluated by the process of exflagellation (extrusion of rapidly waving flagellum-like microgametes from the infected erythrocyte). The activation of female gametocytes is evaluated based on the specific expression of the P. falciparum s25 (*Pf*s25) protein at the surface of the female activated gametes (46). Male P. falciparum gametocytes exflagellate when activated, causing movement of the surrounding red blood cells (RBCs) in the medium. By detecting these changes in cells’ positions, we were able to detect activated male gametes. Female P. falciparum gametocytes round up when activated, and the *Pf*s25 protein becomes widely distributed in the membrane of the gamete. Using a monoclonal antibody against this protein, we were able to specifically detect activated female gametes. A dual gamete formation assay was performed, and the results are shown in Table 2.

**TABLE 2:** *In vitro* IC_50_s of (−)-*R*,*S*-dehydroemetine and (−)-*S*,*S*-dehydroisoemetine against male and female gametocytes in P. falciparum strain NF54

Compound	IC_50_ (μM) for^*a*^:			
	Male gametocytes		Female gametocytes	
	Avg	SD	Avg	SD
(−)-*R*,*S*-dehydroemetine	0.43	0.02	1.04	0.02
(−)-*S*,*S*-dehydroisoemetine	>10		>10	

a *In vitro* IC_50_s of (−)-*R*,*S*-dehydroemetine and (−)-*S*,*S*-dehydroisoemetine in P. falciparum dual gamete formation assay.

### hERG channel inhibition assay.

Dose-related cardiovascular side effects observed following treatment of amoebiasis with emetine included ECG changes such as T-wave inversion, prolongation of QT interval, and widening of the QRS complex and PR interval. Hypotension, tachycardia, and precordial pain were also observed (25). Stoppage of treatment resulted in complete recovery of cardiovascular functions. Cardiac microscopic examination revealed a separation of muscle fibers and destruction of myocardial fibers but an absence of inflammatory cells, leading to the interpretation that the myocarditis is toxic rather than inflammatory in origin. In another study on 32 patients, pain was noted at the injection site, along with ECG abnormalities, myalgia, muscle weakness, and increased levels of serum creatinine phosphatase (47). In a study on guinea pigs by Schwartz and Herrero (27), it was observed that dehydroemetine was excreted faster than emetine. It was also postulated that reduced cardiotoxicity of dehydroemetine could be due to decreased tissue affinity to the heart in comparison to that of emetine (25).

In a resting cardiac cell, the concentration of K^+^ ions is high intracellularly, which creates a chemical gradient for K^+^ ions to diffuse out of the cells. A subunit of the rapid delayed rectifier potassium ion channel is involved in the cardiac repolarization (48). It is encoded by the hERG gene (human ether-a-go-go-related gene). Since emetine is known to affect the movements of Na^+^, K^+^, and Ca^2+^ ions, 2,3-dehydroemetine is also thought to affect ion permeability. hERG channel inhibition assays were therefore carried out to determine potential influences on the cardiotoxicity previously observed with emetine therapy.

### Data analysis of hERG channel inhibition assay results.

For each replicate, the hERG response was calculated using the following equation: % hERG response = post-compound-application current (nA)/pre-compound-application current (nA) × 100. The % hERG response was plotted against the concentration of the test compound, and where concentration-dependent inhibition was observed, the data were fitted to the following equation and an IC_50_ value calculated:

$$y=\frac{y_{\text{max }}-y_{\text{min }}}{1+{\left(\frac{{\text{IC}}_{50}}{x}\right)}^{s}}+y_{\text{min }}$$

where *y* is the hERG response, *y*_max_ is the mean of the 100% vehicle control response, *y*_min_ is the mean of the 0% vehicle control response, *x* is the concentration, IC_50_ is the concentration required to inhibit current by 50%, and *s* is the Hill slope.

(−)-*R*,*S*-dehydroemetine has an IC_50_ of 19.3 μM for the hERG channel, whereas (−)-*S*,*S*-dehydroisoemetine has an IC_50_ of 2.99 μM (Table 3). The IC_50_ of quinidine, which was used as a positive control, is 1.99 μM. The selectivity index was calculated as IC_50_ hERG/IC_50_ parasites. Thus, it was found that, with a selectivity index (SI) of over 271, (−)-*R*,*S*-dehydroemetine is not an hERG channel inhibitor, but (−)-*S*,*S*-dehydroisoemetine is a potent inhibitor (selectivity index = 1.48).

**TABLE 3:** hERG channel inhibition assay results for (−)-*R*,*S*-dehydroemetine and (−)-*S*,*S*-dehydroisoemetine^*a*^

Compound	Mean % hERG channel inhibition at concentration (μM) of:							IC_50_ (μM)
	0	0.016	0.08	0.4	2	10	50	
(−)-*R*,*S*-dehydroemetine	0	7.79	6.34	12.7	7.21	39.4	61.4	19.3
(−)-*S*,*S*-dehydroisoemetine	0	8.13	4.61	9.33	36	70.7	96.9	2.99

a This experiment was outsourced to Cyprotex, UK. Each value is the mean of triplicate values.

### Staining with rhodamine 123 for measurement of mitochondrial membrane potential.

**FIG 9:**
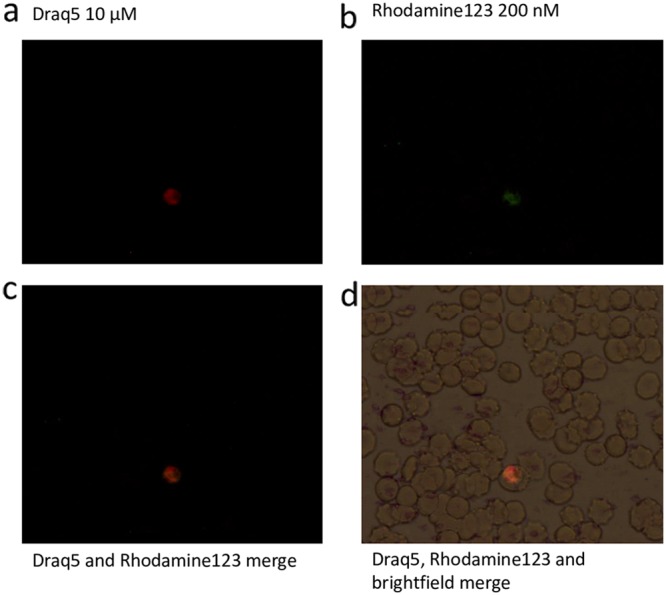
Staining of parasites with rhodamine 123 and Draq5. Merge with bright-field image shows localization of the dye within the parasite. P. falciparum K1 strain trophozoites stained with 10 μM Draq5 (a) and 200 nM rhodamine 123 (b), Draq5 and rhodamine merge (c), and Draq5, rhodamine 123, and brightfield merge (d). Visualized under fluorescence microscope at ×100 magnification.

Changes in mitochondrial membrane potential were measured using rhodamine 123 (excitation wavelength, 511 nm; emission wavelength, 534 nm), a membrane-permeating cationic fluorescent dye (49), which accumulates by electrostatic attraction in the mitochondria because of its negative transmembrane potential. A change in the dye’s concentration in the mitochondria is caused by a depolarization event and can be visualized as a shift in the fluorescence intensity of rhodamine 123 (50). Draq5 (excitation wavelength, 647 nm; emission wavelength, >665 nm) is a far-red fluorescent DNA dye which is cell permeating and was used to distinguish the parasites in flow cytometry in the allophycocyanin (APC)-Cy7-A channel. SYBR green was not preferred for this experiment as it emits in the same channel as rhodamine 123 and would result in the overlap of emission signals. Fluorescence microscopy was used to visualize the localization of rhodamine 123 within the cytoplasm of the parasites (Fig. 9).

**FIG 10:**
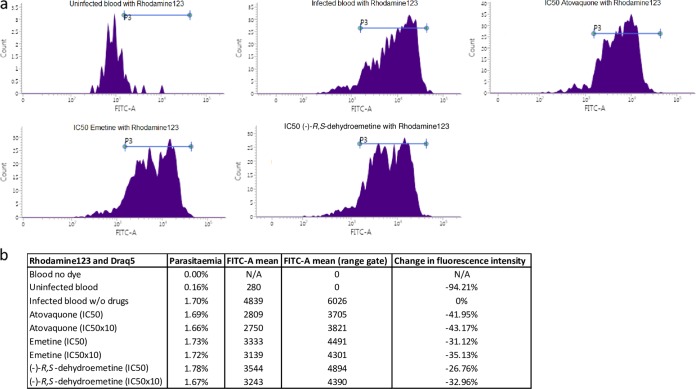
Disruption of mitochondrial membrane potential. Changes in mitochondrial membrane potential observed on treatment with IC_50_s of atovaquone, emetine, and (−)-*R*,*S*-dehydroemetine. (a) Graphical representation of changes in fluorescence intensity on application of drugs at IC_50_s. (b) Table showing changes in fluorescence intensity (FITC-A mean) on application of drugs at IC_50_s and 10× IC_50_s. FITC-A mean value of infected blood without drugs was considered to be the base value (0), and FITC-A mean values of the compounds are represented as percentages of deviation from the base value.

A decrease in fluorescence intensity measured in the fluorescein isothiocyanate (FITC)-A channel indicates a loss of mitochondrial membrane potential. Atovaquone, a known mitochondrial inhibitor, was used as a control. Shifts in fluorescence intensity were observed after treatment with all three compounds at IC_50_s and 10× IC_50_s. Emetine and (−)-*R*,*S*-dehydroemetine showed shifts in the fluorescence intensity of rhodamine 123 in a direction similar to that of atovaquone, indicating a possible mitochondrial effect (Fig. 10). Atovaquone produced 41.95% and 43.17% changes in mean fluorescence intensity at the IC_50_ and 10× IC_50_, respectively, emetine produced changes of 31.12% to 35.13% at the IC_50_ and 10× IC_50_, respectively, in a direction similar to that of atovaquone, and (−)-*R*,*S*-dehydroemetine produced changes of 26.76% to 32.98% at the IC_50_ and 10× IC_50_, respectively, in a direction similar to that of atovaquone (Fig. 10a).

### Validation of the CalcuSyn assay for drug interaction analysis for malaria.

The CalcuSyn method has been validated in our laboratory as a useful tool for antimalarial drug interaction analysis (38). To establish the robustness of the method, the known synergistic drug combination atovaquone and proguanil was used. Triplicate samples were analyzed after 72 h using the SYBR green-based fluorescence plate reader method. In accordance with published literature, CalcuSyn predicted strong synergism between the two drugs.

Following validation of the CalcuSyn software using the atovaquone-proguanil combination, the interactions of (−)-*R*,*S*-dehydroemetine–atovaquone and (−)-*R*,*S*-dehydroemetine–proguanil were studied. The doses used for each compound were based on the known 50% effective dose (ED_50_) values, which served as the midpoints for 2-fold, constant-ratio dose series as shown in Table 4.

**TABLE 4:** Dose series used for the combination of existing antimalarials with (−)-*R*,*S*-dehydroemetine

Level	Value for^*a*^:		
	(−)-*R*,*S*-dehydroemetine (nM)	Atovaquone (nM)	Proguanil (μM)
A	12.5	0.5	1.75
B	25	1	3.5
C	50	2	7
D	100	4	14
E	200	8	28
F	400	16	56
G	800	32	112

a The ratios for combination with (−)-*R,S*-dehydroemetine were 1:25 for atovaquone and 140:1 for proguanil.

### CalcuSyn-based drug interaction analysis of the atovaquone-proguanil combination.

**FIG 11:**
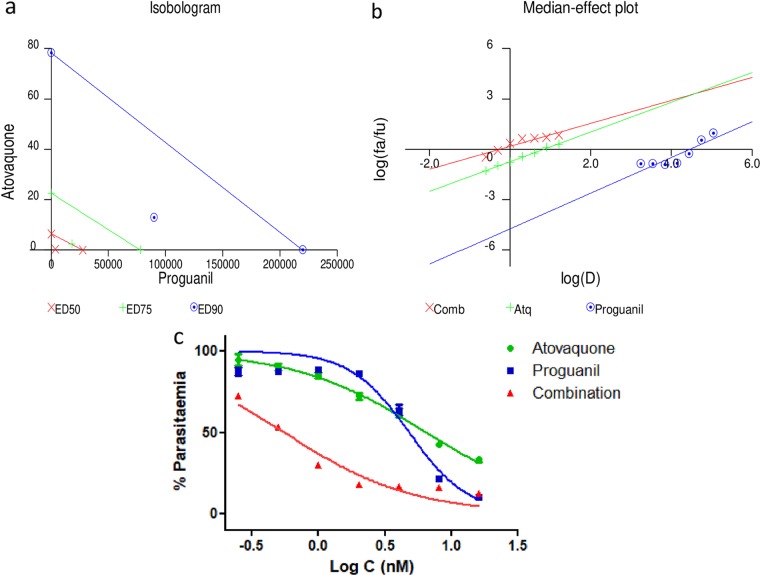
CalcuSyn-based drug interactivity analysis for atovaquone and proguanil. CalcuSyn-based median effect plot (a), isobologram (b), and dose-effect curve analyzed through GraphPad Prism (c) for drug interactivity between atovaquone and proguanil. The combination of atovaquone and proguanil (1:7,000) was found to be strongly synergistic at the IC_50_ (combination index [CI] = 0.21) and IC_75_ (CI = 0.34) and synergistic at the IC_90_ (CI = 0.57). fa, fraction affected; fu, fraction unaffected.

The CalcuSyn-based analysis of the drug interactivity between atovaquone and proguanil was carried out using a constant-ratio combination of 1:7,000. The output included a dose-effect curve and a median-effect plot in addition to the combination index (CI) and an isobologram plot, to figuratively depict the compounds’ potency and conformity to the mass action law (Fig. 11). Specifically, CI values of 0.20, 0.34, and 0.57 at the ED_50_, ED_75_, and ED_90_ levels (Table 5), respectively, were obtained, inferring strong synergism at the ED_50_ and synergism at the ED_75_ and ED_90_. Good correlation coefficients of the median-effect plot were reported for atovaquone (*r* = 0.99), proguanil (*r* = 0.89), and their combination (*r* = 0.93), inferring good conformity to the mass action law.

**TABLE 5:** CalcuSyn-based drug interaction analysis^*a*^

Drug combination	CI at:			*r*
	ED_50_	ED_75_	ED_90_	
Atovaquone and proguanil (1:7,000)	0.21	0.34	0.57	0.93
Atovaquone and (−)-*R*,*S*-dehydroemetine (1:25)	0.88	0.88	0.89	0.96
Proguanil and (−)-*R*,*S*-dehydroemetine (140:1)	0.67	1.04	1.62	0.95

a The combination ratios and CalcuSyn determined the combination index (CI) values for the assays performed. The *r* value represents the linear correlation coefficient for the median effect plot and indicates conformity to the mass action law.

### CalcuSyn-based drug interaction analysis for the (−)-*R*,*S*-dehydroemetine–atovaquone combination.

**FIG 12:**
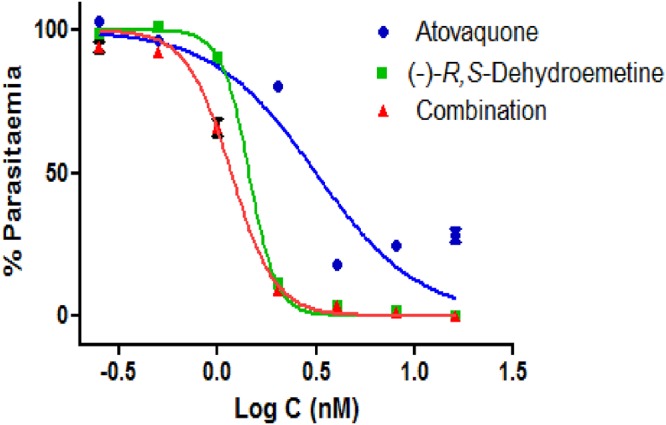
CalcuSyn-based drug interactivity analysis for (−)-*R*,*S*-dehydroemetine–atovaquone combination. Dose-effect curve analyzed through GraphPad Prism for drug interactivity between (−)-*R*,*S*-dehydroemetine and atovaquone. The combination of atovaquone and (−)-*R*,*S*-dehydroemetine (1:25) was found to display slight synergy at the IC_50_ (CI = 0.88), IC_75_ (CI = 0.88), and IC_90_ (CI = 0.89).

The CalcuSyn-based analysis of the drug interactivity between (−)-*R*,*S*-dehydroemetine and atovaquone was done using a constant-ratio combination of 25:1 (Fig. 12). Specifically, CI values of 0.88, 0.88, and 0.89 at the ED_50_, ED_75_, and ED_90_ levels, respectively, were obtained, inferring slight synergism at all measurement points (Table 5). Good correlation coefficients of the median-effect plot were reported for atovaquone (*r* = 0.94), (−)-*R*,*S*-dehydroemetine (*r* = 0.94), and the combination (*r* = 0.96).

### CalcuSyn-based drug interaction analysis for the (−)-*R*,*S*-dehydroemetine–proguanil combination.

**FIG 13:**
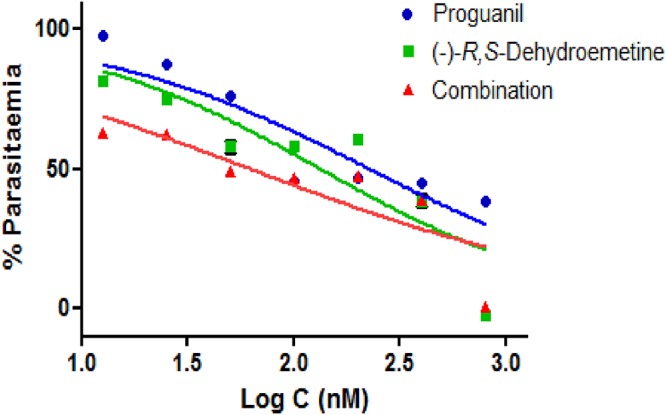
CalcuSyn drug interactivity analysis for (−)-*R*,*S*-dehydroemetine–proguanil combination. Dose-effect curve analyzed through GraphPad Prism for drug interactivity between proguanil and (−)-*R*,*S*-dehydroemetine. The combination of proguanil and (−)-*R*,*S*-dehydroemetine (140:1) was found to be synergistic at the IC_50_ (CI = 0.67), nearly additive at the IC_75_ (CI = 1.04), and antagonistic at the IC_90_ (CI = 1.62).

The CalcuSyn-based analysis of the drug interactivity between (−)-*R*,*S*-dehydroemetine and proguanil was done using a constant-ratio combination of 140:1 (Fig. 13). Specifically, CI values of 0.67, 1.04, and 1.62 at the ED_50_, ED_75_, and ED_90_ levels, respectively, were obtained, implying synergism at the ED_50_, a nearly additive effect at the ED_75_, and antagonism at the ED_90_ (Table 5). Good correlation coefficients of the median-effect plot were obtained for proguanil (*r* = 0.90), (−)-*R*,*S*-dehydroemetine (*r* = 0.86), and the combination (*r* = 0.95), suggesting good conformity to the mass action law.

After 72 h of incubation, the (−)-*R*,*S*-dehydroemetine–atovaquone interaction was found to show slight synergism at all inhibitory levels analyzed. For the (−)-*R*,*S*-dehydroemetine–proguanil combination, the ED_50_ level of inhibition was classified as synergism, the ED_75_ as nearly additive, and the ED_90_ as antagonism.

## DISCUSSION

We report here the nanomolar antimalarial efficacy of the synthetic emetine analogue (−)-*R*,*S*-dehydroemetine in the multidrug-resistant K1 strain of Plasmodium falciparum (IC_50_ of 71.03 ± 6.1 nM). The clinical use of emetine dihydrochloride as an antiamoebic drug was superseded by the better-tolerated synthetic analogue 2,3-dehydroemetine in the 1970s. The isomer (−)-*S*,*S*-dehydroisoemetine was found to be less potent, with an IC_50_ of 2.07 ± 0.26 μM. Molecular modeling suggests that the greater potency of (−)-*R*,*S*-dehydroemetine is linked to its ability to mimic the interactions of emetine with the E site of the *Pf*40S subunit more fully than the (−)-*S*,*S*-dehydroisoemetine isomer does. The improvements in the dose-dependent cardiotoxicity previously reported for 2,3-dehydroemetine in comparison to that of emetine dihydrochloride was linked to decreased affinity to cardiac myocytes and increased clearance from the body (27). We propose that the very significant differential *in vitro* activities of emetine dihydrochloride in Entamoeba histolytica (IC_50_ of 26.8 ± 1.27 μM) and Plasmodium falciparum (IC_50_ of 47 ± 2.1 nM) (13, 14) would mean that the dose-related toxicity profile for its repositioned use will be different.

Analysis done by GSK showed that in asexual blood stages, the compounds exhibited no cross-resistance issues. Mosquito vector dynamics, the number of people with peripheral gametocytemia in the population, and the infectiousness of circulating gametocytes to mosquitoes determine the transmission of malaria. Interrupting transmission is an important aspect of preventing malaria in areas of endemicity, and there has been renewed interest in compounds preventing the formation of gametocytes. (−)-*R*,*S*-dehydroemetine was found to be gametocidal. It displayed activity against both male and female gametes. Thus, it also has the potential to block the transmission of malaria.

The selectivity indices calculated using MTT assays in HepG2 cell lines were similar for emetine and its analogues. It is important to note that the low selectivity indices were expected, given the documented anticancer properties of emetine (51). This could explain the low LD_50_s obtained on HepG2 lines, as these are rapidly multiplying cancer cell lines. The selective advantage of (−)-*R*,*S*-dehydroemetine for cardiotoxic effects is linked to its faster elimination from the body, and hence, the appropriate toxicity investigation to define this would be the use of *in vivo* animal models. It is imperative to progress this work in this direction. It was also clearly established that the cardiac toxicity for (−)-*R*, *S*-dehydroemetine was not effected through the hERG channel. Our observations suggest that emetine and its two synthetic analogues produce changes in the mitochondrial membrane potential at their IC_50_s, indicating a possible multimodal mechanism of action.

The failure of monotherapy has been unambiguously demonstrated in malaria, and hence, there is forceful insistence by WHO on the use of artemisinin-based combination therapy (ACT) as a policy standard. In addition to more potent therapeutic efficacies, other benefits of combination regimes include decreased toxicity, favorable synergistic interactions, and most significantly, the potential to impede or delay the onset of resistance. The drug interaction analysis presented here emphasizes a route to further dose reduction to minimize toxicity. (−)-*R*,*S*-dehydroemetine was found to exhibit synergistic activity with proguanil and display slight synergism with atovaquone.

Emetine and its analogues constitute a pleiotropic group of natural product-derived compounds that could potentially enhance the depleted antimalarial armamentarium should a catastrophic gap in the drug market occur as a result of the spread of resistance to frontline antimalarials. The work presented here provides strong justification for further optimization, particularly for use in a hospital setting in cerebral malaria, where appropriate monitoring could obviate progression of the reversible cardiotoxicity previously reported. Their hepatic concentration ability could indeed have favorable consequences for the treatment of Plasmodium vivax infections. The history of antimalarial chemotherapy has been largely reliant on natural product-derived leads. We provide here a significant body of evidence to progress and further optimize a previously overlooked, potent, and affordable natural product compound.

## MATERIALS AND METHODS

### Culture of Plasmodium falciparum.

RPMI 1640 medium containing 25 mM HEPES and 0.3 g/liter l-glutamine (Gibco; Life Technologies, UK) supplemented with 2.5 g sterile filtered AlbuMax (Sigma, UK), 2.5 ml hypoxanthine (Sigma, UK), 2.5 ml 40% glucose (anhydrous dextrose; Fisher Scientific, UK), and 0.5 ml gentamicin (Sigma, UK) was used for the culture of erythrocytic-stage strain K1 P. falciparum parasites (gifted by John Hyde, University of Manchester, United Kingdom; original source, Thai-K1 clone) under a 5% CO_2_, 5% O_2_, and 90% N_2_ gas mixture (BOC Limited, UK) at 37°C. All routine culture methods were consistent with those described in reference 52.

O positive human blood (purchased from NHS Blood Bank, Manchester, UK) was used routinely to maintain the parasites. Continuous cultures were maintained at 5% hematocrit. Since synchronous parasite development is observed in natural hosts and this synchrony is lost quite rapidly in *in vitro* cultures, sorbitol was used to keep the parasites in tight synchrony. In brief, the parasitized blood pellet was resuspended 1:10 in 5% sorbitol (prepared in distilled water and filtered using a 0.22-μm-porosity Millipore filter), incubated at room temperature for 5 min, and then centrifuged at 3,000 rpm for 5 min. The supernatant was removed, and complete medium was used to wash the pellet 3 times before setting up a new culture.

### Synthesis of (−)-2,3-dehydroemetine.

**FIG 14:**
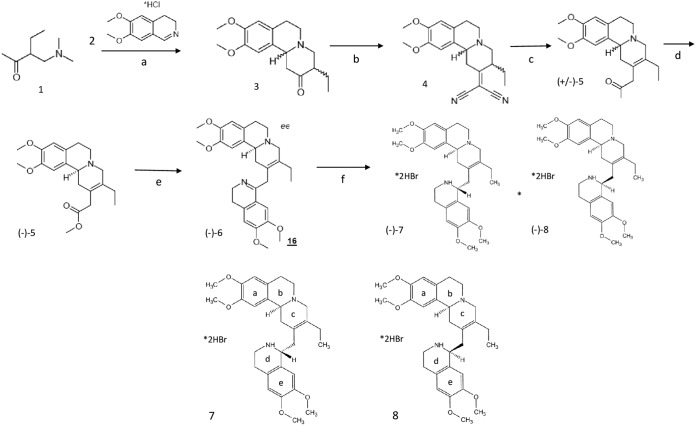
Synthesis of 2, 3-dehydroemetine. Conditions were as follows. (a) (i) Iodomethane, ethanol, room temperature (RT), 24 h, (ii) compound 2, KOAc, reflux, 3 h, 72%; (b) malononitrile, ammonium acetate/acetic acid, toluene, reflux, 2.5 h, 69%; (c) (i) 20% HCl, reflux, 5 h, (ii) methanolic HCl, RT, 18 h, 45%; (d) (+)-dibenzoyl tartrate, methanol, two recrystallizations, 25%; (e) (i) 3 M HCl, reflux, 90 min, (ii) 3,4-dimethoxyphenethylamine, xylenes, reflux, 18 h, (iii) POCl_3_, benzene, reflux, 1 h, 28%; (f) NaBH_4_, methanol, RT, 1 h, 81% of a 1:1 diastereomeric mixture. Compound 7, (*S*)-2-((*R*)-6,7-dimethoxy-1,2,3,4-tetrahydro-isoquinolin-1-ylmethyl)-3-ethyl-9,10-dimethoxy-1,6,7,11b-tetrahydro-4H-pyrido[2,1-a]isoquinoline * dihydrobromide. Compound 8, (S)-2-((S)-6,7-dimethoxy-1,2,3,4-tetrahydro-isoquinolin-1-ylmethyl)-3-ethyl-9,10-dimethoxy-1,6,7,11b-tetrahydro-4H-pyrido[2,1-a]isoquinoline * dihydrobromide.

(−)-2,3-Dehydroemetine was synthesized according to the methods in the established literature (16, 53). The synthesis was outsourced to Chiroblock GMBH, Germany. Patent literature previously published by chemists at Hoffmann-La Roche did not include modern analytical data, such as mass spectrometry or nuclear magnetic resonance (NMR) (53). The methodology adopted for the synthesis of (−)-*R*,*S*-dehydroemetine (compound 7) used in this study followed the published patent, with minor modifications, as depicted in Fig. 14.

The two compounds synthesized were (−)-*R*,*S*-dehydroemetine, compound 7, and (−)-*S*,*S*-dehydroisoemetine, compound 8. Briefly, the Mannich base, compound 1, was quaternized and reacted with the imine, compound 2, giving the piperidinone, compound 3, as an approximately 5:1 mixture of diastereomers that were not separated. Knoevenagel condensation with malononitrile led to unsaturated dinitrile, compound 4, in a 69% yield. Hydrolysis and decarboxylation, with concomitant alekene isomerization, gave compound 5 after methylation. Resolution with (+)-dibenzoyl-*d*-tartaric acid gave the homochiral compound, compound (−)-5. Hydrolysis and amidation with homoveratrylamine, followed by Bischler-Napieralski cyclization, led to a dihydroisoquinoline derivative, compound 6, also known as 2-dehydro-*O*-methyl psychotine. The cyclic imine was reduced with sodium borohydride in methanol to give a 1:1 mixture of the desired 2,3-dehydroemetine, compound 7, and 2-dehydroisoemetine dihydrobromide, compound 8, separated by fractional crystallization. The purity of (−)-*R*,*S*-dehydroemetine obtained through this method was 85%, with the balance consisting of the diastereomer, compound 8.

### Molecular modeling.

In preparation for predicting the bound poses of emetine, (−)-*R*,*S*-dehydroemetine, and (−)-*S*,*S*-dehydroisoemetine, the cryo-EM structure of the P. falciparum 80S ribosome bound to emetine dihydrochloride was obtained from the Protein Data Bank (PDB code 3J7A) (16). We note that this cryo-EM structure features the enantiomer of emetine rather than emetine itself; therefore, an initial three-dimensional (3-D) structure of emetine was obtained from the OMEGA-generated conformer in the PubChem database (54). Subsequently, emetine derivatives (−)-*R*,*S*-dehydroemetine and (−)-*S*,*S*-dehydroisoemetine were constructed using the software package MOE (55). Prior to *in silico* docking, crystallographic water molecules and a magnesium ion were removed (the latter was within 13 Å of the active site and was found to unduly influence docking solutions). Hydrogens were assigned consistent with physiological pH. Docking of ligands to the *Pf*40S subunit was performed using MOE-Dock (2016; Chemical Computing Group, Inc., Montreal, QC, Canada). The binding site region was defined using the cryo-EM ligand location. Ligand placement used the Triangle Matcher protocol. Internal flexibility was permitted for ligands but not ribosome. Poses were scored via the London dG scoring function. While this docking protocol was able to reproduce well the cryo-EM pose of the emetine stereoisomer bound to the *Pf*40S subunit (not shown), please see the comment in Results regarding this ligand’s structure.

### Drug preparation.

Emetine dihydrochloride was obtained from Sigma-Aldrich, UK. The dehydroemetine analogues were synthesized as described in the established literature (16; scheme 1 in reference 53). Derivatives were validated using NMR, high-performance liquid chromatography (HPLC), and Fourier transform infrared spectroscopy (FTIR). The synthesis process was outsourced to Chiroblock GMBH, Germany. Drug stock solutions were prepared in dimethyl sulfoxide (DMSO) in accordance with the manufacturer’s instructions, and the primary stock concentration of 5 mM was aliquoted and stored at –20°C until further use. Serial dilutions of the working solution using complete medium were made for the experiments.

### Experimental determination of IC_50_ to test activity against the K1 strain of P. falciparum.

Refined dose ranges were selected for emetine dihydrochloride (12.5 nM to 200 nM), (−)-*R*,*S*-dehydroemetine (12.5 nM to 200 nM), and (−)-*S*,*S*-dehydroisoemetine (0.625 μM to 10 μM) to permit accurate calculation of their IC_50_s against the K1 strain of P. falciparum. Ring stage parasites were diluted to 0.5 to 1% parasitemia, 2.5% hematocrit (in a 96-well-plate format, 200-μl final well volume) and treated for 72 h. Dose-response parasitemia was determined using the SYBR green-based flow cytometer or plate reader method previously optimized in the laboratory (14).

### SYBR green staining of erythrocytic-stage P. falciparum parasites for flow cytometry.

Following drug efficacy experiments, 150-μl amounts from the control and drug-treated wells on a 96-well plate were transferred to microcentrifuge tubes, and samples were washed once with phosphate-buffered saline (PBS). The supernatant was removed, and samples were incubated in the dark at room temperature for 20 min following the addition of 1 ml of 5× SYBR green solution (prepared by adding 5 μl of 10,000× SYBR green to 10 ml PBS). After staining, the samples were centrifuged for 1 min at 14,000 rpm and the supernatant was discarded. Samples were resuspended in 250 μl of 0.37% formaldehyde fixation solution prepared by diluting 36.5% formaldehyde (Sigma, UK) with PBS to the specified final concentration. The samples were placed in the refrigerator and incubated at 4°C for 15 min. Subsequently, PBS was used to wash the samples 3 times, and finally, the samples were suspended in 1 ml PBS. Parasitemia was determined by measuring SYBR green fluorescence using the FITC channel of the BD FACSVerse flow cytometer system (blue laser; excitation laser line, 488 nm, with excitation maximum of 494 nm and emission maximum of 520 nM) and cell size (forward scatter [FSC-A]). Fifty thousand events were recorded, in three replicates for each. Fluorescence events in drug-treated samples were compared with those in their infected and uninfected blood counterparts and gated accordingly to obtain the percentages of parasitemia.

### Time course analysis through IC_50_ speed assay using unsynchronized cultures of Plasmodium falciparum.

The IC_50_ speed assay was used to determine the speeds of action of the emetine analogues. Unsynchronized cultures of K1 strain P. falciparum were used, and parasites were grown in the presence of (−)-*R*,*S*-dehydroemetine and (−)-*S*,*S*-dehydroisoemetine for three incubation periods of 24 h, 48 h, and 72 h. The assays were analyzed by determining SYBR green fluorescence as described above.

### Stage-specific profiling of (−)-*R*,*S*-dehydroemetine and (−)-*S*,*S*-dehydroisoemetine using synchronized cultures of Plasmodium falciparum.

Parasite cultures with ≥80% trophozoites and ≥80% rings were obtained by synchronization with 5% sorbitol (42). The cultures were synchronized twice, at 0 h and 31 h, to obtain young rings which were up to 3 h old. To obtain early schizont stages, the cultures were synchronized twice, with the second synchronization being 6 to 8 h after the first. Each synchronous stage was incubated for 24 h at 37°C on a 96-well microtiter plate with a 2-fold serial dilution of the drugs ranging from 1.6- to 100-fold IC_50_ of each drug. The plates were washed 4 times after the incubation in order to dilute the drug concentration by >1,000-fold. The plates were incubated for another 24 h at 37°C, following which SYBR green staining was used to read the plates as described above.

### MTT assay for cell cytotoxicity against HepG2 cells.

HepG2 cells (University of Salford stocks, purchased from American Type Culture Collection [ATCC], USA), were grown in medium consisting of RPMI 1640, 2 mM l-glutamine, HEPES, 10% fetal bovine serum, 1% nonessential amino acids, and 1% penicillin-streptomycin. Cells were calculated on a hemocytometer to make a new cell-medium solution to a concentration of 5,000 cells/100 μl, and 100 μl of this new cell solution was added into each well of the 96-well plate. After 24 h of incubation, 100-μl amounts of the drug prepared in the medium were added to the wells. This was repeated in triplicate, and the plate was incubated for a further 48 h (56). After the incubation period, 50 μl of MTT solution was added to each well and incubated for 3 h. The liquid in each well was aspirated carefully, 200 μl of DMSO was added to each well, and the results were read on the Ascent plate reader.

### Derivation of dose-response curves and IC_50_ values.

The results for the infected-blood controls were set at 100% for normalization, and percentages of parasitemia for drug-treated samples were calculated relative to the results for the infected controls. For IC_50_ values, GraphPad Prism 5.0 was used to further process the data. IC_50_ values were calculated using nonlinear regression (GraphPad Prism 5.0) by using log-transformed drug concentrations plotted against the dose response. The log(inhibitor) versus normalized response-variable slope option was used for IC_50_ calculation. Emetine dihydrochloride was used as a control drug to validate the method.

### Determination of cross-resistance through IC_50_ determination in multidrug-resistant P. falciparum strains.

The three strains used for the cross-resistance assay were 3D7A, Dd2, and W2, based on their resistance profiles; the assay was carried out by GSK, Tres Cantos. Strain 3D7A is chloroquine sensitive, strain Dd2 is resistant to chloroquine, mefloquine, and pyrimethamine (57), and strain W2 is resistant to chloroquine, quinine, pyrimethamine, cycloguanil, and sulfadoxine (from the Malaria Research and Reference Reagent Resource Center [MR4]) (58). A culture of red blood cells (RBCs) parasitized by the corresponding strain (0.5% parasitemia, 2% hematocrit) in RPMI 1640, 5% AlbuMax, and 5 μM hypoxanthine was exposed to 9 dilutions (3-fold serial dilutions) of the compound starting at 5 μM. One hundred microliters of culture volume was plated in 96-well flat-bottom microtiter plates with 0.5 μl drug (200× stock in DMSO). Plates were incubated for 24 h at 37°C, 5% CO_2_, 5% O_2_, 90% N_2_. Next, 3H-hypoxanthine was added and plates were incubated for another 24-h period. After that, parasites were harvested on a glass fiber filter using a TomTec cell harvester 96. Filters were dried, and melt-on scintillator sheets were used to determine the incorporation of 3H-hypoxanthine. Radioactivity was measured using a MicroBeta counter. Data were normalized by incorporating the results for the positive control (parasitized red blood cells without drug). IC_50_ values were determined using the Grafit 7 program (41).

### Determination of transmission-blocking potential through *in vitro* inhibition of gamete activation.

Asexual cultures of P. falciparum strain NF54 parasites were used to seed gametocyte cultures at 0.5% parasitemia, 4% hematocrit in a 50-ml total volume under 3% O_2_, 5% CO_2_, 92% N_2_ gas. Culture medium (RPMI, 25 mM HEPES, 50 mg/liter hypoxanthine, 2 g/liter NaHCO_3_ without l-glutamine plus 5% human serum and 5% AlbuMax) was replaced daily for 14 days. At day 14, the concentration of nonpurified cultures was adjusted to plate 700,000 total cells per well in each 384-well plate. The test drugs were then added in 2-fold serial dilutions starting at 10 μM (10 μM to 0.00976 μM) and incubated for 48 h at 37°C (3% O_2_, 5% CO_2_, 92% N_2_). DMSO was used as the negative control, and thiostrepton as the positive control. Activation was performed with ookinete medium (same RPMI base used for culture but supplemented with 50 μM xanthurenic acid) supplemented with anti-*Pf*s25-Cy3 antibody at a final concentration of 1/2,000 (from 1 mg/ml stock). Plates were analyzed to detect exflagellation centers. “Triggered” cultures were then incubated (protected from light) at 26°C for 24 h (in a thermoregulated incubator). Then, plates were analyzed to detect female activated gametes. Activation of male gametes was detected based on light changes provoked by flagellar movements that caused movement of surrounding cells. A 10-frame video was taken and then analyzed to determine these changes in cell position based on changes in pixels. Then, the script determined where exflagellation centers were located, also based on the size and intensity of light changes. Activation of female gametes was based on detection of fluorescent Cy3-anti *Pf*s25 antibody (as the primary parameter), followed by a selection of events according to their size and roundness and the intensity of the fluorescence. Both measurements were performed using an automated inverted Ti-E Nikon microscope and JOBS software. Analysis of images and videos was performed with the ICY program. The IC_50_ values were determined using Microsoft Excel and GraphPad.

### hERG channel inhibition (IC_50_ determination) assay protocol.

The experiment to test the potential of emetine, (−)-*R*,*S*-dehydroemetine, and (−)-*S*,*S*-dehydroisoemetine to inhibit the hERG channel was outsourced to Cyprotex, UK. One-hundred-microliter amounts of a 20 mM concentration of each of the three compounds in DMSO was provided to Cyprotex. Compound dilutions were prepared by diluting a DMSO solution (10 mM default) of the test compound into DMSO using a 5-fold dilution scheme, followed by dilution into extracellular buffer such that the final concentrations tested were typically 0.008, 0.04, 0.2, 1, 5, and 25 μM (final DMSO concentration, 0.25%). Chinese Hamster ovary cells expressing the hERG potassium channel were dispensed into 384-well planar arrays, and hERG tail currents were measured by whole-cell voltage clamping. Amphotericin B was used as a perforating agent that was circulated underneath the PatchPlate to gain electrical access to the cell. The pre-compound-application hERG current was measured. Emetine, (−)-*R*,*S*-dehydroemetine, and (−)-*S*,*S*-dehydroisoemetine in ranges of concentrations were then added to the cells, and a second recording of the hERG current was made. The test compound was left in contact with the cells for 300 s before currents were recorded. Quinidine, an established hERG inhibitor, was included as a positive control, and a vehicle control (0.25% DMSO) as the negative control. All buffers, cell suspensions, and drug compound solutions were at room temperature. The percentage of change in hERG current was measured and used to calculate an IC_50_ value.

Each concentration was tested in 4 replicate wells on the PatchPlate (maximum of 24 data points). Filters were applied to ensure that only acceptable cells were used to assess hERG inhibition. The cell must maintain a seal resistance of greater than 50 MΩ and a pre-compound-application current of at least 0.1 nA and ensure cell stability between pre- and post-compound-application measurements.

### Staining with rhodamine 123 for mitochondrial membrane potential disruption.

Rhodamine 123 and Draq5 were used to observe the effects of emetine and its two synthetic analogues on mitochondrial membrane potential. Rhodamine 123 is a mitochondrial-specific dye which emits in the FITC channel in flow cytometry, whereas Draq5 stains the DNA and emits in the APC-Cy7-A channel. These two dyes were chosen to perform the experiment as there is very minimal overlap in their emission signals. A synchronized culture of P. falciparum strain K1 at the trophozoite stage was incubated with 1 ml of 200 nM rhodamine 123 for 1 h, followed by a further incubation with 100 μl of 10 μM Draq5 for 20 min. Smears prepared after the incubation were immediately viewed under the fluorescence microscope at ×100 magnification to visualize the localization of the dyes inside the parasite.

To test the effects of emetine and its analogue on mitochondria, synchronized cultures of P. falciparum strain K1 at the trophozoite stage were incubated at 2.5% hematocrit (in a 96-well-plate format with a final well volume of 200 μl) for 2 h with IC_50_s and 10× IC_50_s of atovaquone, emetine hydrochloride, and (−)-*R*,*S*-dehydroemetine. Compounds were then washed away by centrifuging the cultures, and a pellet was prepared for each drug concentration. Pellets were then incubated with 1 ml of 200 nM rhodamine 123 for 1 h, followed by a further incubation with 100 μl of 5 μM Draq5 for 20 min. After a wash with PBS, the experiment was read with a flow cytometer on the FITC channel. Loss of mitochondrial membrane potential was indicated by a decrease in fluorescence intensity.

### Drug interaction analysis for (−)-*R*,*S*-dehydroemetine.

Primary stock solutions of (−)-*R*,*S*-dehydroemetine were prepared as described above. For the experimental setup, the primary stock solutions were further diluted with complete medium to give final test concentrations. A dose range of 0.125 to 8 times the ED_50_ was made by 2-fold serial dilutions for atovaquone, proguanil, and (−)-*R*,*S*-dehydroemetine. For atovaquone, the doses ranged from 0.25 nM to 16 nM for combination with proguanil and from 0.5 nM to 32 nM for combination with (−)-*R*,*S*-dehydroemetine. For proguanil, the doses ranged from 1.75 μM to 112 μM. For (−)-*R*,*S*-dehydroemetine, the doses ranged from 12.5 nM to 800 nM. At each level, the compounds were coadministered; for example, the ED_50_ of atovaquone was combined with the ED_50_ of (−)-*R*,*S*-dehydroemetine, 2 times the ED_50_ of atovaquone was combined with 2 times the ED_50_ of (−)-*R*,*S*-dehydroemetine, and so forth. Parasites were treated at ring stage and incubated for 72 h in a 96-well-plate format. The SYBR green-based plate reader method was used to determine drug susceptibility. The data were analyzed for the median effect using CalcuSyn software (Biosoft) by converting triplicate data to an averaged percentage. The *r* values are also reported for all sets of data. The *r* value represents the linear correlation coefficient for the median-effect plot and indicates conformity to the mass action law. The CalcuSyn software generates the CI over a range of fraction-affected (*f_a_*) levels at different growth inhibition percentages. The interpretation of CI was done in accordance with the classification presented in Table 6 (37).

**TABLE 6:** Classification of synergism or antagonism using CI values generated by the Chou-Talalay method (CalcuSyn manual, Biosoft [38])

Range of CI values	Symbol	Description
<0.1	+++++	Very strong synergism
0.1–0.3	++++	Strong synergism
0.3–0.7	+++	Synergism
0.7–0.85	++	Moderate synergism
0.85–0.90	+	Slight synergism
0.90–1.10	+	Nearly additive
1.10–1.20	−	Slight antagonism
1.20–1.45	−−	Moderate antagonism
1.45–3.3	−−−	Antagonism
3.3–10	−−−−	Strong antagonism
>10	−−−−−	Very strong antagonism

### Ethics statement.

For routine malaria culture, anonymized whole-blood packs deemed unfit/outdated for clinical use were purchased from the NHS Blood Bank at Plymouth Grove, Manchester, UK. For experiments carried out in GlaxoSmithKline, Diseases of the Developing World Medicines Development Campus, Tres Cantos, Spain, the human biological samples were sourced ethically and their research use was in accord with the terms of the informed consents under an IRB/EC-approved protocol.
